# Absolute Eosinophil Count as a Corroborative Marker for Sepsis at ICU Admission: An Observational Study

**DOI:** 10.7759/cureus.117288

**Published:** 2026-10-01

**Authors:** Chathur Agnivesh Balaji, Arun Divakar, MGK Pillai

**Affiliations:** 1 Department of General Medicine, Amrita School of Medicine, Kochi, IND; 2 Department of General Medicine, Amrita Institute of Medical Sciences and Research Centre, Kochi, IND

**Keywords:** absolute eosinophil count, eosinopenia, qsofa, qsofa-e, sepsis, sofa

## Abstract

Background

Among critically ill patients, sepsis continues to be a major cause of morbidity and mortality. Timely action requires an early diagnosis, but current screening methods have limitations. One commonly available hematological metric that has gained attention as a possible indicator of systemic illness is the absolute eosinophil count (AEC). The usefulness of AEC as a corroborative marker for sepsis diagnosis at intensive care unit (ICU) admission was studied in this study, along with its correlation with scores on the Sequential Organ Failure Assessment (SOFA) and quick Sequential Organ Failure Assessment (qSOFA).

Methods

This observational study included 76 adult patients admitted to the ICU with suspected infection between February 7, 2025, and March 31, 2026. Sepsis was classified according to the Third International Consensus Definitions for Sepsis and Septic Shock (Sepsis-3) criteria. SOFA and qSOFA scores were calculated, and AEC was assessed at ICU admission. Receiver operating characteristic (ROC) curve analysis was used to derive an optimal AEC cutoff for identifying sepsis. Eosinopenia at the study-derived cutoff was incorporated as one additional point into qSOFA to construct an exploratory modified score, qSOFA-E. Correlations between AEC and SOFA/qSOFA scores were assessed using Spearman's rank correlation coefficient.

Results

Among the 76 patients, 38 (50%) fulfilled the Sepsis-3 criteria, 18 (23.7%) developed septic shock, and 7 (9.2%) died during hospitalization. The optimal AEC cutoff for identifying sepsis was ≤152.5 cells/µL, with an area under the ROC curve (AUC) of 0.904 (95% CI: 0.828-0.979; p<0.001). At this cutoff, AEC demonstrated 92.11% sensitivity, 84.21% specificity, and 88.16% diagnostic accuracy. AEC showed significant inverse correlations with day-1 SOFA (r=-0.585, p<0.001), day-3 SOFA (r=-0.443, p=0.005), day-1 qSOFA (r=-0.664, p<0.001), and day-3 qSOFA (r=-0.534, p=0.001). In this study cohort, qSOFA-E demonstrated higher sensitivity and diagnostic accuracy than qSOFA alone (92.11% vs. 65.79% sensitivity; 96.05% vs. 82.89% accuracy).

Conclusions

AEC was associated with the presence of sepsis and with higher SOFA and qSOFA scores in this ICU cohort. Incorporation of study-derived eosinopenia into qSOFA was associated with higher diagnostic sensitivity and accuracy within the study population. These findings support further investigation of AEC as an adjunctive marker and qSOFA-E as a proposed exploratory modification. Larger multicenter studies with independent validation are required before these findings can be generalized to broader clinical practice.

## Introduction

Despite major advancements in organ support techniques, critical care monitoring, and antimicrobial therapy, sepsis continues to be one of the main causes of morbidity and mortality among critically ill patients. It still has a significant global impact on intensive care unit (ICU) admissions and mortality, highlighting the significance of prompt identification and treatment [1].

In a significant improvement from the previous definitions based on the systemic inflammatory response syndrome (SIRS), the Third International Consensus Definitions for Sepsis and Septic Shock (Sepsis-3) define sepsis as life-threatening organ failure brought on by a dysregulated host response to infection. Both predictive and clinical applicability have improved with this updated classification, which highlights organ failure as the hallmark of clinically severe sepsis [2]. A popular tool for determining the degree of organ malfunction and forecasting clinical outcomes in sepsis patients is the Sequential Organ Failure Assessment (SOFA) score. However, its routine use necessitates a number of laboratory indicators, such as platelet counts, serum bilirubin and creatinine levels, arterial blood gas analysis, and vasopressor needs, which may limit its applicability during early assessment or in settings with limited resources [2]. The quick Sequential Organ Failure Assessment (qSOFA) score was developed as a streamlined screening tool to enable quick bedside examination. Despite the ease of use of qSOFA, a number of validation studies have revealed inadequate sensitivity for the early detection of sepsis, especially in patients with developing organ dysfunction [3,4].

Delays in starting antimicrobial medication and supportive measures are linked to higher morbidity and death; therefore, early detection of sepsis is crucial [5]. Despite being widely utilized in clinical practice, biomarkers including procalcitonin and C-reactive protein (CRP) have limited availability in some healthcare settings, inconsistent specificity, and delayed reaction kinetics [6]. Therefore, low-cost, easily accessible, and quickly achievable biomarkers that can supplement current clinical assessment techniques are still needed.

As a common component of a complete blood count, the absolute eosinophil count (AEC) has become recognized as a possible indicator of physiological stress and systemic illness. Eosinopenia, a rapid drop in circulating eosinophil levels linked to acute infections, is mediated by immunological and neuroendocrine responses during systemic inflammation [7]. Eosinopenia may help differentiate infectious from non-infectious inflammatory disorders and may be correlated with illness severity and prognosis in critically sick patients, according to prior research [7-10]. Additionally, eosinophils' immunomodulatory capabilities and their part in host defense mechanisms outside of allergy and parasitic illnesses are becoming more widely acknowledged [11].

Despite these observations, the diagnostic and prognostic significance of eosinophil count in sepsis, particularly its relationship with established severity assessment tools such as SOFA and qSOFA scores, remains inadequately characterized. Therefore, the present study was undertaken to evaluate the role of AEC as an adjunctive marker for the early identification of sepsis at ICU admission and to assess its association with SOFA and qSOFA scores.

Primary objective

The main aim of this study was to evaluate the utility of AEC as a corroborative marker in the diagnosis of sepsis on ICU admission.

Secondary objectives

The secondary objectives were to study the role of AEC as a prognostic marker to assess the morbidity and mortality in sepsis, to find the correlation of SOFA and qSOFA scores with AEC, and to compare the sensitivity and specificity of qSOFA and qSOFA-E scores.

## Materials and methods

Study design and setting

This single-center observational study was conducted in the ICU of Amrita School of Medicine, Kochi, Kerala, India. Adult patients aged ≥18 years who were admitted to the ICU with suspected infection were enrolled consecutively during the study period from February 7, 2025, to March 31, 2026. A total of 76 patients were included in the final analysis.

Ethical permission and consent

The study was approved by the Institutional Ethics Committee of the participating institution (EC/NEW/INST/2023/KL/0379). Written informed consent was obtained from all participants or their legally authorized representatives before enrollment. Patient confidentiality was maintained throughout the study. No additional investigations or interventions were performed exclusively for research purposes. The study was not registered with the Clinical Trials Registry-India (CTRI), as it was an observational study without allocation of participants to an intervention.

Inclusion criteria

Adult patients (≥18 years) admitted to the ICU with suspected infection were eligible for inclusion. Patients without sepsis were not excluded. All eligible patients admitted with suspected infection were included irrespective of whether they subsequently fulfilled the Sepsis-3 criteria. Thus, both patients with and without sepsis were included in the final study population.

Exclusion criteria

Patients with conditions known to independently alter eosinophil counts were excluded. These included patients with a documented past medical history or previous physician diagnosis of bronchial asthma or chronic obstructive pulmonary disease (COPD), hematological malignancies, autoimmune disorders, eosinophilic granulomatous diseases, connective tissue disorders, and anaphylaxis, as well as those who had received systemic corticosteroid therapy within the preceding 48 hours. Asthma and COPD were identified from the documented past medical history and previous physician diagnosis. Spirometry was not performed during ICU admission for the purpose of applying the exclusion criteria.

Sample size calculation

$n = \frac{Z{(\alpha/2)}^{2} \times p(\%) \times q(\%)}{d(\%)^{2}}$

where p is the observed prevalence, q = 100 - p, d is the margin of error, and Z(α/2)² is the ordinate of standard normal distribution at α% level of significance.

Calculations

p (Prevalence of ICU admission acquired due to sepsis) = 0.06 [12], q = 0.94, d = 0.06, Z(α/2) = 1.96 at α = 5% level of significance

$n = \frac{(1.96)^2 \times 0.06 \times 0.94}{(0.06)^2} = 60.16$

Hence, the minimum sample size required for the present study was 60, which was increased to 70 considering the attrition. During the study period, we were able to recruit 76 patients.

Definition

Sepsis was defined according to Sepsis-3 as life-threatening organ dysfunction caused by a dysregulated host response to infection. Operationally, sepsis was considered present when suspected or documented infection was accompanied by an acute increase in SOFA score of ≥2 points from baseline. In patients without known pre-existing organ dysfunction, the baseline SOFA score was considered to be 0. The Sepsis-3 classification was used as the reference standard for evaluating the diagnostic performance of AEC, qSOFA, and qSOFA-E in the present study [2].

Data collection

Demographic and clinical data were recorded at ICU admission. Clinical parameters including systolic blood pressure, respiratory rate, and mental status were documented. Routine laboratory investigations included complete blood count with differential leukocyte count, performed using an automated hematology analyzer. AEC was obtained directly from the complete blood count report.

SOFA and qSOFA assessment

The SOFA score was calculated using six organ-system domains: respiratory, coagulation, liver, cardiovascular, central nervous system, and renal function. Each domain was assigned a score from 0 to 4 according to the severity of dysfunction, resulting in a total score ranging from 0 to 24. Higher scores indicated greater organ dysfunction [13]. SOFA was calculated at ICU admission (day 1) and repeated on day 3. The day-1 SOFA score was used for Sepsis-3 classification, while the change between day 1 and day 3 was used to assess short-term disease progression.

The qSOFA score was calculated at ICU admission using three clinical variables: systolic blood pressure ≤100 mmHg, respiratory rate ≥22 breaths/minute, and altered mental status (Glasgow Coma Scale score <15). One point was assigned for each criterion, producing a total score ranging from 0 to 3. A qSOFA score of ≥2 was considered positive [3].

Modified qSOFA-E score

An exploratory modified qSOFA score incorporating eosinopenia (qSOFA-E) was developed in the present study. Receiver operating characteristic (ROC) curve analysis was performed using Sepsis-3 classification as the reference standard to assess the ability of day-1 AEC to discriminate between patients with and without sepsis. The optimal AEC cutoff was determined from the ROC curve using the maximum Youden index (sensitivity + specificity − 1). The resulting cutoff was 152.5 cells/µL. An additional point was assigned for eosinopenia, defined as an AEC ≤152.5 cells/µL. Thus, qSOFA-E consisted of the three conventional qSOFA variables plus one additional point for eosinopenia. Both qSOFA and qSOFA-E were evaluated using a threshold of ≥2 for comparison with Sepsis-3 classification. Because the AEC cutoff was derived and evaluated within the same study cohort, qSOFA-E was considered an exploratory modification rather than a validated clinical scoring system.

Statistical analysis

IBM SPSS Statistics Version 26.0 (IBM Corp., Armonk, NY, USA) was used for statistical analysis. Continuous variables were reported as mean ± standard deviation or median with interquartile range, depending on the distribution of the data. Categorical variables were presented as frequencies and percentages. Sepsis-3 classification was used as the reference standard for assessment of the diagnostic performance of AEC, qSOFA, and qSOFA-E. ROC curve analysis was performed to evaluate the discriminatory ability of day-1 AEC and to determine the optimal AEC cutoff using the maximum Youden index. Sensitivity, specificity, positive predictive value, negative predictive value, and diagnostic accuracy were calculated. Spearman's rank correlation coefficient was used to assess the relationship between AEC and SOFA/qSOFA scores. The association of qSOFA and qSOFA-E score categories (<2 vs. ≥2) with Sepsis-3 status was assessed using Pearson's chi-square test. No missing data were present for the variables included in the analysis, and therefore no missing-data imputation was required. A p-value of <0.05 was considered statistically significant.

## Results

Among the 76 study participants, 47 (61.8%) were males and 29 (38.2%) were females. Sepsis was diagnosed in 38 (50.0%) patients, while 38 (50.0%) did not fulfill the Sepsis-3 criteria. Eighteen patients (23.7% of the total study population) developed septic shock; all 18 patients with septic shock were among the 38 patients who fulfilled the Sepsis-3 criteria. The overall in-hospital mortality was 9.2% (7/76). Among patients fulfilling the Sepsis-3 criteria (n=38), urinary tract infection was the most common etiology (44.7%), followed by lower respiratory tract infection (28.9%), bacteremia (21.1%), and other causes (5.3%), as shown in Table 1.

**Table 1:** Demographic and clinical characteristics of the study population (n = 76) *Etiology of sepsis was calculated among patients fulfilling Sepsis-3 diagnostic criteria (n=38). All 18 patients classified as having septic shock were included within the Sepsis-3-positive group. Sepsis status was determined using the Sepsis-3 definition, requiring suspected or documented infection with an acute increase in SOFA score of ≥2 points from baseline. UTI, urinary tract infection; LRTI, lower respiratory tract infection

Variable	Category	Frequency (n)	Percentage (%)
Gender	Male	47	61.8
	Female	29	38.2
Sepsis status	Yes	38	50.0
	No	38	50.0
Septic shock	Yes	18	23.7
	No	58	76.3
Mortality	Yes	7	9.2
	No	69	90.8
Etiology of sepsis*	UTI	17	44.73
	LRTI	11	28.95
	Bacteremia	8	21.05
	Others	2	5.26

In this study, 41 patients had an AEC of ≤152.5 cells/µL and 35 patients had an AEC of >152.5 cells/µL. Among patients with an AEC of ≤152.5 cells/µL, 35 had sepsis and 6 did not. Among patients with an AEC of >152.5 cells/µL, 3 had sepsis and 32 did not (Table 2).

**Table 2:** Diagnostic performance of day-1 absolute eosinophil count for the diagnosis of sepsis

AEC (cells/µL)	Sepsis	No sepsis	Total
≤152.5	35	6	41
>152.5	3	32	35
Total	38	38	76

ROC analysis demonstrated excellent diagnostic performance of both day-1 AEC and day-1 SOFA score for identifying sepsis. The optimal day-1 AEC cutoff value was ≤152.5 cells/µL, yielding an AUC of 0.904 (95% CI: 0.828-0.979; p < 0.001). At this threshold, AEC showed 92.11% sensitivity, 84.21% specificity, 85.37% positive predictive value, 91.43% negative predictive value, and an overall diagnostic accuracy of 88.16%. Patients with AEC ≤ 152.5 cells/µL had a significantly higher prevalence of sepsis compared with those having higher eosinophil counts (85.4% vs. 8.6%; p < 0.001). The day-1 SOFA score demonstrated an even higher discriminative ability with an AUC of 0.985 (95% CI: 0.969-1.000; p < 0.001), indicating near-perfect diagnostic performance (Table 3, Figure 1).

**Table 3:** Diagnostic performance of day-1 AEC and day-1 SOFA score for the diagnosis of sepsis (n=76) Diagnostic performance was evaluated using ROC curve analysis with Sepsis-3 classification as the reference standard. The optimal day-1 AEC cutoff of ≤152.5 cells/µL was determined from ROC analysis using the maximum Youden index. AEC values at or below this cutoff were considered test-positive for sepsis. For SOFA, a score of ≥2 was considered positive for clinically significant organ dysfunction in accordance with the Sepsis-3 framework. AUC, area under the ROC curve; CI, confidence interval; PPV, positive predictive value; NPV, negative predictive value; AEC, absolute eosinophil count; SOFA, Sequential Organ Failure Assessment

Diagnostic variable	Cut-off	AUC (95% CI)	Sensitivity, % (95% CI)	Specificity, % (95% CI)	PPV, % (95% CI)	NPV, % (95% CI)	Accuracy, % (95% CI)
Day-1 AEC	≤152.5 cells/µL	0.904 (0.828-0.979)	92.11 (78.62-98.34)	84.21 (68.75-93.98)	85.37 (70.83-94.43)	91.43 (76.94-98.20)	88.16 (78.71-94.44)
Day-1 SOFA	≥2	0.985 (0.969-1.000)	94.74 (82.25-99.36)	97.37 (86.19-99.93)	97.30 (85.84-99.93)	94.87 (82.68-99.37)	96.05 (88.89-99.18)

**Figure 1:**
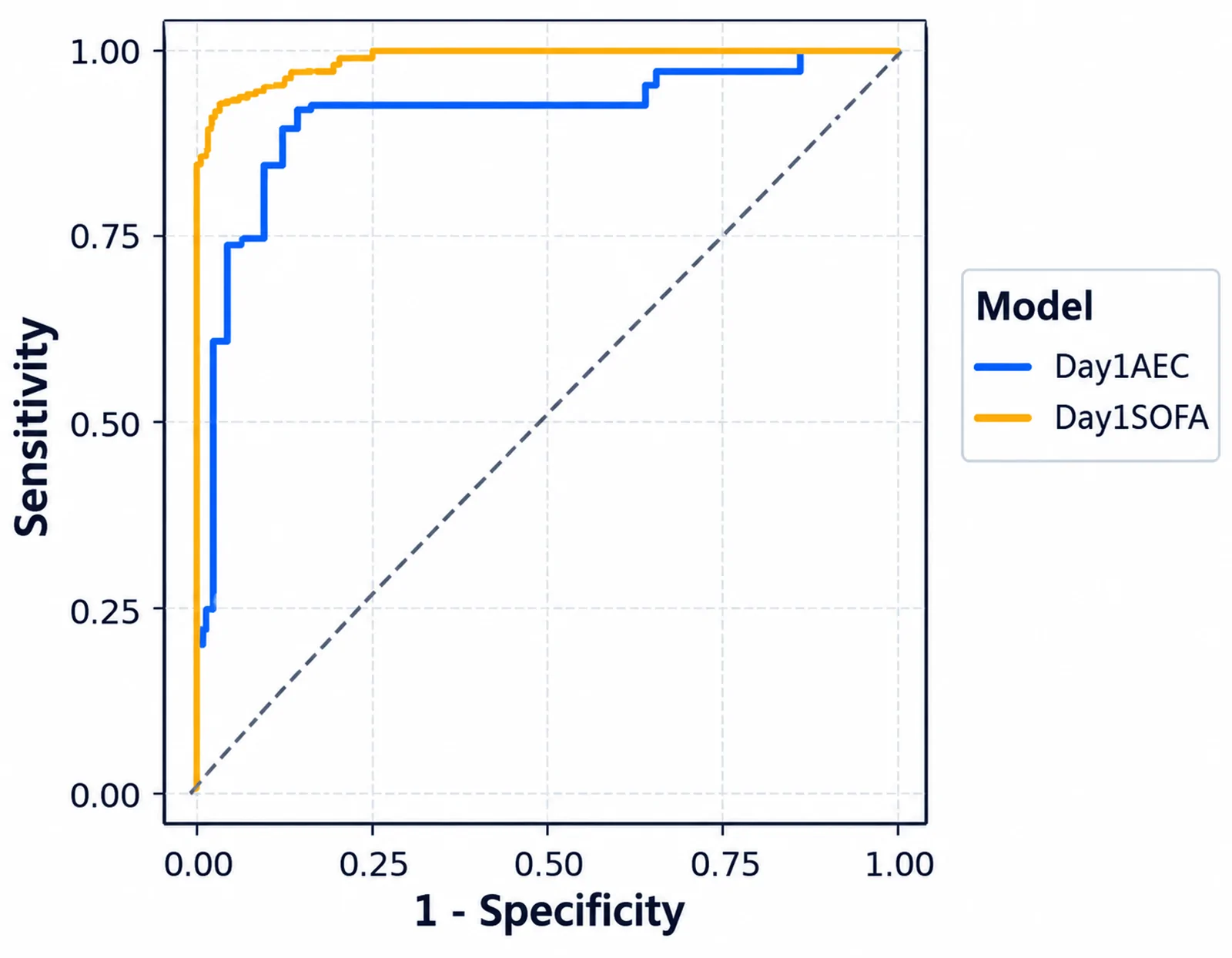
ROC curves of day-1 AEC and day-1 SOFA for the diagnosis of sepsis Day-1 AEC AUC = 0.904 (95% CI: 0.828–0.979); Day-1 SOFA AUC = 0.985 (95% CI: 0.969–1.000) ROC, receiver operating characteristic; AEC, absolute eosinophil count; SOFA, Sequential Organ Failure Assessment; AUC, area under the ROC curve

There was a significant inverse correlation between AEC and both SOFA and qSOFA scores on days 1 and 3 (Table 4). Lower AEC was consistently associated with higher severity scores, indicating greater organ dysfunction and clinical deterioration. The strongest correlation was observed between day-1 AEC and day-1 qSOFA score (r=−0.664, p < 0.001), suggesting that eosinopenia is a reliable marker of disease severity in patients with sepsis.

**Table 4:** Correlation of AEC with SOFA and qSOFA scores Spearman's rank correlation test was used to assess the correlation between AEC and SOFA/qSOFA scores. The correlation coefficient (r) and corresponding p-values are presented. AEC, absolute eosinophil count; SOFA, Sequential Organ Failure Assessment; qSOFA, quick Sequential Organ Failure Assessment

Correlation	Day	Correlation coefficient (r)	P-value
AEC vs SOFA	Day 1	-0.585	<0.001
AEC vs SOFA	Day 3	-0.443	0.005
AEC vs qSOFA	Day 1	-0.664	<0.001
AEC vs qSOFA	Day 3	-0.534	0.001

The inclusion of eosinopenia in qSOFA to construct qSOFA-E was associated with higher sensitivity and diagnostic accuracy within the study cohort. Using a threshold of ≥2, qSOFA-E demonstrated a sensitivity of 92.11% and diagnostic accuracy of 96.05%, compared with 65.79% and 82.89%, respectively, for conventional qSOFA. Both scores demonstrated 100% specificity in this cohort (Table 5). 

**Table 5:** Diagnostic performance of qSOFA and qSOFA-E for sepsis qSOFA and qSOFA-E were assessed against Sepsis-3 classification as the reference standard. A score ≥2 was considered positive. qSOFA-E was calculated by adding one point for eosinopenia (AEC ≤ 152.5 cells/µL) to the qSOFA score. The P-value represents the association between score category (<2 vs ≥2) and Sepsis-3 status, assessed using Pearson's chi-square test. CI, confidence interval; qSOFA, quick Sequential Organ Failure Assessment; qSOFA-E, quick Sequential Organ Failure Assessment-eosinophil; AEC, absolute eosinophil count

Variable	qSOFA	qSOFA-E
Score ≥2, n/N (%)	25/76 (32.9)	35/76 (46.1)
Score <2, n/N (%)	51/76 (67.1)	41/76 (53.9)
Sepsis among patients with score ≥2, n/N (%)	25/25 (100.0)	35/35 (100.0)
Sepsis among patients with score <2, n/N (%)	13/51 (25.5)	3/41 (7.3)
Sensitivity, % (95% CI)	65.79 (48.65–80.37)	92.11 (78.62–98.34)
Specificity, % (95% CI)	100.00 (90.75–100.00)	100.00 (90.75–100.00)
Positive predictive value, % (95% CI)	100.00 (86.28–100.00)	100.00 (90.00–100.00)
Negative predictive value, % (95% CI)	74.51 (60.37–85.67)	92.68 (80.08–98.46)
Accuracy, % (95% CI)	82.89 (72.53–90.57)	96.05 (88.89–99.18)
χ² statistic	37.25	64.88
P-value	<0.001	<0.001

## Discussion

Due to its complex etiology and varied clinical presentation, sepsis continues to be a major cause of morbidity and mortality in critically ill patients, and early diagnosis remains difficult. Sepsis is not a uniform inflammatory process but rather a dysregulated host response to infection that causes potentially fatal organ failure [1]. Early immunological changes may occur before obvious clinical deterioration, adding to the diagnostic challenge during the initial evaluation of critically ill patients. The SOFA score has become an established measure of organ dysfunction and a predictor of mortality in sepsis [14]. However, its routine use during early evaluation may be limited by its reliance on multiple laboratory parameters, which may not be readily available in emergency departments or resource-limited settings.

The qSOFA score was developed as a simplified bedside tool that does not require laboratory investigations. Despite its ease of use, several validation studies have demonstrated limited sensitivity of qSOFA for early identification of sepsis, particularly in patients with evolving organ dysfunction [15]. This has prompted interest in additional biomarkers that could complement bedside clinical assessment without substantially increasing complexity or cost.

Biomarkers such as procalcitonin and CRP are commonly used in the assessment of sepsis; however, their limited availability in some healthcare settings, variable specificity, and delayed changes during the course of illness may restrict their utility [6,16]. Consequently, routinely available hematological parameters have attracted interest as inexpensive and rapidly obtainable adjunctive markers.

Eosinophils, traditionally associated with allergic and parasitic conditions, are now recognized as immunologically active cells involved in tissue homeostasis, immune regulation, and cytokine signaling. During acute infection and systemic inflammation, circulating eosinophil levels may decrease because of reduced bone marrow release, stress-induced corticosteroid responses, and migration of eosinophils to inflammatory sites [17]. This phenomenon, termed eosinopenia, has therefore been investigated as a potential marker of acute infection and systemic inflammatory states.

The present study evaluated the association between AEC and established severity assessment tools and examined its potential role as a corroborative diagnostic marker for sepsis. In our cohort, eosinopenia at ICU admission demonstrated good diagnostic performance and was significantly associated with sepsis. These findings are consistent with previous clinical studies and meta-analyses reporting an association between eosinopenia and sepsis [10,14,18].

A major finding of the present study was the significant inverse correlation between AEC and SOFA score at both initial and subsequent assessment. Lower eosinophil counts were associated with higher SOFA scores, indicating greater organ dysfunction. This association may reflect the relationship between eosinopenia and the systemic inflammatory and immunological changes accompanying severe infection. Similar relationships between eosinopenia and disease severity have been reported previously [14,19]. However, because of the observational design of the present study, these associations should not be interpreted as evidence of a causal relationship.

AEC also demonstrated a significant inverse correlation with qSOFA scores. This finding suggests that lower eosinophil counts were associated with the physiological abnormalities captured by qSOFA in patients with suspected infection. Based on this observation, eosinopenia was incorporated into qSOFA to construct the modified qSOFA-E score. In the present cohort, qSOFA-E demonstrated higher sensitivity and diagnostic accuracy than conventional qSOFA for identifying patients fulfilling Sepsis-3 criteria.

The potential clinical relevance of incorporating AEC into bedside assessment lies in its accessibility. AEC is routinely available as part of a complete blood count and does not require additional specialized testing. This contrasts with biomarkers such as procalcitonin, which may require greater laboratory resources and incur additional costs [6,14]. Therefore, AEC may represent a practical adjunctive parameter for sepsis assessment, particularly in resource-limited settings. This is consistent with the emphasis of international guidelines on early recognition and prompt initiation of appropriate therapy in sepsis [20].

The present study extends previous work by exploring the incorporation of eosinopenia into an established bedside scoring system rather than evaluating eosinopenia solely as an isolated diagnostic or prognostic marker [12,16]. The findings are consistent with the concept that sepsis assessment benefits from integration of clinical and laboratory information rather than reliance on a single biomarker [21]. The observed increase in diagnostic performance with qSOFA-E therefore provides a basis for further investigation of combined physiological and hematological assessment.

An important methodological consideration is the use of Sepsis-3 classification as the reference standard. Sepsis-3 defines sepsis using an acute increase in SOFA score of ≥2 points in the setting of suspected or documented infection. Consequently, SOFA forms part of the reference standard against which AEC and qSOFA-E were evaluated. This introduces an element of reference-standard incorporation or circularity when interpreting the relationship between AEC, SOFA, and Sepsis-3 classification. Therefore, the observed associations and diagnostic performance should be interpreted with this consideration in mind.

Another important limitation is that the AEC cutoff of 152.5 cells/µL was derived using ROC analysis from the same cohort in which its diagnostic performance was assessed. An independent validation cohort was not available. Consequently, the reported performance of the cutoff and the resulting qSOFA-E score may be optimistic and could reflect characteristics specific to this study population. Independent validation in larger and clinically diverse populations is therefore required before the cutoff or qSOFA-E can be considered for routine clinical application.

Overall, the findings of the present study support the potential role of AEC as an adjunctive marker in the assessment of sepsis and provide preliminary evidence for incorporating eosinopenia into a bedside scoring approach. However, qSOFA-E should currently be regarded as an exploratory modification rather than a validated clinical scoring system. Larger multicenter studies should evaluate the reproducibility of the AEC cutoff, validate qSOFA-E in independent populations, assess additional biomarkers and clinical variables, and determine whether such an approach improves clinically relevant outcomes.

Limitations

This study has several limitations. First, it was conducted at a single center, which may limit the generalizability of the findings to other healthcare settings and patient populations. Second, although the final sample exceeded the revised minimum sample-size requirement, the sample size remained relatively small for evaluation of a newly proposed scoring modification and may have limited the precision of diagnostic-performance estimates. Third, the AEC cutoff of 152.5 cells/µL was derived using ROC analysis within the same cohort in which it was evaluated. No independent validation cohort was available; therefore, the reported diagnostic performance may be subject to optimism or overfitting. External validation in larger multicenter cohorts is required. Fourth, Sepsis-3 classification in this study incorporated an acute increase in SOFA score of ≥2 points. Therefore, evaluation of AEC against Sepsis-3 and comparison with SOFA involve an element of reference-standard incorporation or circularity, which should be considered when interpreting the diagnostic associations. Fifth, the study did not evaluate long-term outcomes such as 28-day mortality or ICU length of stay. Outcome assessment was limited to in-hospital mortality and short-term clinical progression based on the change in SOFA score from day 1 to day 3. Finally, qSOFA-E incorporated only eosinophil count in addition to the conventional qSOFA variables and did not evaluate other potentially informative biomarkers such as procalcitonin, serum lactate, or other hematological parameters. Larger multicenter studies using independent validation cohorts and multivariable approaches are required to determine the incremental diagnostic value of AEC and to establish whether incorporation of eosinopenia into bedside scoring systems provides reproducible clinical benefit.

## Conclusions

In this single-center observational cohort of adults admitted to the ICU with suspected infection, lower AEC was associated with the presence of sepsis and with higher SOFA and qSOFA scores. An AEC cutoff of ≤152.5 cells/µL demonstrated good diagnostic performance for identifying sepsis within the study cohort.

Incorporation of this study-derived eosinopenia criterion into qSOFA was associated with higher sensitivity and diagnostic accuracy than conventional qSOFA in the present population. These findings support the potential role of AEC as an adjunctive marker in the assessment of patients with suspected sepsis and provide preliminary evidence for further investigation of qSOFA-E.

However, the findings should be considered exploratory because of the small, single-center study design, the use of SOFA within the Sepsis-3 reference definition, and the absence of independent validation of the AEC cutoff and qSOFA-E. Larger multicenter studies with independent validation are required to confirm these findings, establish reproducible cutoff values, and determine the clinical utility of incorporating AEC into sepsis screening approaches.
